# *Alocasia cucullata* Exhibits Strong Antitumor Effect In Vivo by Activating Antitumor Immunity

**DOI:** 10.1371/journal.pone.0075328

**Published:** 2013-09-25

**Authors:** Qiuxian Peng, Hongbing Cai, Xuegang Sun, Xin Li, Zhixian Mo, Jue Shi

**Affiliations:** 1 School of Chinese Medicine, Southern Medical University, Guangzhou, China; 2 Centre for Quantitative Systems Biology, Department of Physics and Department of Biology, Hong Kong Baptist University, Hong Kong, China; Baylor College of Medicine, United States of America

## Abstract

Chinese herbal medicines have long been used to treat various illnesses by modulating the human immune response. In this study, we investigate the immuno-modulating effect and antitumor activity of *Alocasia Cucullata* (AC), a Chinese herb traditionally used to treat infection and cancer. We found that the whole water extract of AC roots could significantly attenuate tumor growth in mouse tumor models. The median survival time of the AC-treated mice was 43 days, 16 days longer than that of the control group. Moreover, the AC-treated mice showed substantially higher induction of key antitumor cytokines, such as IL-2, IFN-γ, and TNF-α, indicating that AC may exert antitumor effect by activating antitumor immunity. To further pinpoint the cellular and molecular mechanism of AC, we studied the dose response of a human monocytic cell line, THP-1, to the whole water extract of AC. Treatment of the AC extract significantly induced THP-1 differentiation into macrophage-like cells and the differentiated THP-1 showed expression of specific macrophage surface markers, such as CD11b and CD14, as well as productions of antitumor cytokines, e.g. IFN-γ and TNF-α. Our data thus point to AC as potentially a new, alternative immuno-modulating herbal remedy for anticancer treatment.

## Introduction

Despite decades of extensive effort to develop more effective anticancer chemotherapeutics, the response rate to current cancer chemotherapy in the clinic remains largely unimproved (less than 20%). This is mostly due to the strong heterogeneity in cancer; not only cancer of different disease types is genetically different, even for the same type of cancer, if the tumor is from different patients, the level and spectrum of genetic mutations involved could be highly distinct [1], [2]. It is thus imperative to explore new druggable targets and therapeutic strategies that could tackle the strong genetic heterogeneity of cancer and the resulting patient variability in drug response. One new anticancer strategy that is distinct from traditional cytotoxic regimen and holds clinical potential is immunotherapy [3], [4]. The recent advancement in cancer immunology has revealed a wide variety of roles played by the human immune system that are crucial for oncogenesis, metastasis and treatment outcome, suggesting immunotherapy that reactivates the weakened host immune response against tumors could be a promising anticancer treatment when used alone or in combination with other anticancer chemotherapeutics [5].

Currently available immunotherapy either employs specific cytokines/chemokines to activate *in vivo* the antitumor activities of distinct immune cells or involves activation of immune cells *in vitro* and then transferring the tumor-reactive cells to cancer patients, e.g. adopted T-cell transfer [6], [7]. However, similar to traditional chemotherapy, response variability between disease types and patients as well as treatment-associated side effects are prominent in immunotherapy, posing great challenges for developing cancer immunotherapy with better efficacy and specificity [8], [9]. With a long tradition of treating various diseases through modulating the human immune response, Chinese herbal remedy offers potential new immuno-modulating regimen for cancer treatment. According to Chinese medicinal theories, tumors form likely due to the loss of a healthy balance in the body and therefore are generally treated by strengthening the patients’ immune system. The most widely studied Chinese herbs that are known to enhance immune response include astragalus (*huang qi*), ganoderma (*ling zhi*) and cordyceps (*dong chong xia cao*) [10]–[12].

*Alocasia cucullata* (AC) is a less well-known immuno-modulating herb, traditionally used for detoxification and clearing excessive heat in the body. It is commonly prescribed for treating various infections and also used as an anticancer herb by Chinese medicine practitioners, in particular for treating cervical cancer and breast carcinoma [13]–[15]. AC belongs to the araceae family and the chemical composition of AC was found to consist of mostly polysaccharides (66%) and proteins (7%). The active components that may account for AC’s pharmacological effects possibly include amino acids and polysaccharides [14], which both have been shown to have immuno-stimulatory effects [16], [17]. Although AC consists of potentially immuno-regulating components, it is largely unknown whether AC indeed exerts antitumor effect by activating antitumor immunity. Little has been done to determine the molecular and cellular mechanisms of the anticancer activity of AC, hindering further development of AC as an alternative anticancer remedy for a larger patient population in the clinic.

In this study we investigate the antitumor activity of AC extract and the potential involvement of antitumor immunity in mediating the pharmacological effects of AC, using both mouse tumor models and cultured mammalian cells. Our results showed that the whole water extract of AC roots significantly attenuated tumor growth and prolonged the survival of tumor-bearing mice. The antitumor effect of AC was associated with strong induction of key cytokines *in vivo*, such as IL-2, IFN-γ and TNF-α. Further dose response study with cultured mammalian cell lines showed that AC treatment did not directly trigger cancer cell death. Instead, AC extract activated the differentiation of THP-1, a human monocytic cell line, into macrophage-like cells *in vitro*, with substantial induction of TNF-α and IL-1β. Our findings thus not only establish the strong antitumor activity of AC *in vivo* but also suggest that AC is likely to exert anticancer effect not through direct cytotoxicity on tumor cells, but by activating antitumor immune responses, such as promoting the differentiation of monocytes into active macrophages against the tumor.

## Materials and Methods

### Preparation of whole Water Extract of AC Roots

The *Alocasia cucullata* (AC) plant was grown and harvested in Guangxi province, People’s Republic of China, and was identified by a plant specialist, Prof. Ma (School of Chinese Medicine, Southern Medical University) as AC. The AC roots were first boiled in distilled water at 100°C for 4 hours, and then freeze-dried to a powder form. The AC solution for treating the tumor-bearing mice was adjusted to 1 g (raw herb)/ml. For cell culture experiments, we first dissolved the powder form of AC at 40 mg/ml, and then removed the insoluble grinds and sterilized the solution by filtering it through a 0.20 µm sterile filter. We had measured the level of endotoxin contaminant in the AC solution using an endotoxin assay kit (Genscript) based on the manufacturer’s protocol. The endotoxin level at the highest concentration of AC solution that we used for the cell culture study, i.e. 2 mg/ml, was determined to be 0.146 EU, which was about 0.1% of the endotoxin level that would induce THP-1 differentiation [18]. So endotoxin contamination was negligible in the AC solution that we prepared and used in this study.

### Mouse Tumor Models and Drug Treatment

BALB/c mice (male, 50 in total) weighing 18–20 g, were purchased from the Laboratory Animal Center of Southern Medical University, and housed with free access to water and rodent chow at 20–22°C with 12-hour light-dark cycle. This study and the experimental protocols were approved by the Institutional Animal Care and Use Committee (IACUC) of Southern Medical University. All procedures involving laboratory animals were conducted in accordance with the guidelines of IACUC. For establishing tumor in the mice, 4T1 breast cancer cells (10^6^ cells in 0.1 ml) were inoculated subcutaneously into the mice. Seven days after inoculation, mice were randomly divided into five treatment groups: 1) negative control group (Nctrl) which were treated with saline water; 2) positive control group (Pctrl) which were treated with lentinan; 3) high-dose AC treated group (hAC) which were treated with AC at 16 g/(kg·d), i.e. 16 grams of raw herb per kilogram of mouse weight per day; 4) medium-dose AC treated group (mAC) which were treated with 8 g/(kg·d); and 5) low-dose AC treated group (lAC) which were treated with 4 g/(kg·d). The mice were treated daily by gavage feeding and sacrificed after 21 days.

For assessment of survival time, C57BL/6 mice (half male and half female, n = 20) were obtained from the Laboratory Animal Center of Southern Medical University and inoculated with B16 melanoma cells (1.0×10^6^ cells) subcutaneously. Seven days after the inoculation, the mice were randomly divided into two groups, one treated with saline water (negative control group) and the other with medium dose of AC (i.e. 8 g/(kg·d)). The treatment was administered daily by gavage feeding. The mice were monitored daily for abdominal swelling, ulceration and/or other signs of distress. When it was believed, with the above symptom(s), that a particular mouse would not live, it was sacrificed and the time of death was recorded.

### Evaluation of Tumor and Tumor Tissue Histology

**Figure 1:**
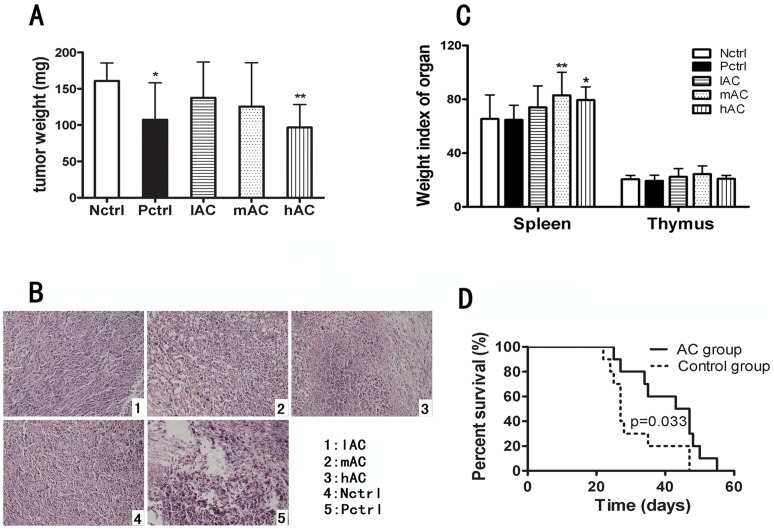
Antitumor effect of AC extract *in vivo.* (A) Comparison of tumor weight in the five treatment groups. Nctrl: negative control group; Pctrl: positive control group; lAC: low-dose AC treatment group; mAC: medium-dose AC treatment group; hAC: high-dose AC treatment group. (B) Morphological structures of tumor tissues from the five treatment groups. Hematoxylin and eosin staining. 200×. (C) Comparison of the spleen and thymus index in the five treatment groups (n = 9 or 10). Data are shown as mean ± S.D. *p<0.05, **p<0.01 *vs* Nctrl group. (D) Cumulative survival curves of the control mice (treated with water) and mice treated daily with medium dose of AC extract (n = 10).

Tumor volume was measured with calipers every four day and evaluated using the standard formula: 0.5×(width)^2^×length. The mice were sacrificed after 21 days. The weights of the tumor, spleen and thymus were measured after the animals were sacrificed. The thymus and spleen indices as shown in Fig. 1C were calculated by normalizing the organ weight to the body weight, using the following formula: (organ weight)/(body weight)×10. For histomorphological analysis, tumor tissues were fixed in phosphate (0.01 mol/L) buffer with 10% formalin (pH 7.4), and then embedded in paraffin. Tissue sections (2 µm thick) were made and stained with hematoxylin and eosin.

### ELISA Analysis of Cytokines

The whole blood was obtained from the Balb/c mice under sterile conditions on the day of sacrifice. The whole blood was then centrifuged at 1000×*g* and 4°C for 30 minutes, and the upper layer containing the serum was collected. The levels of IL-2, INF-γ and TNF-α in the blood serum were determined using ELISA kit (Beyotime, China) according to the manufacturer’s protocol. To measure the levels of TNF-α and IL-1β in the AC-treated THP-1 cells, the culture media was collected and analyzed using the sandwich human ELISA kit (Excell Biology, China) according to manufacturer’s protocol.

### Cell Lines and Cell Culture

The mouse mammary cancer cell line 4T1 (#TCM32) was purchased from the Cell Bank at the Shanghai Institutes for Biological Sciences, Chinese Academy of Sciences. The mouse melanoma cell line, B16, and the human acute monocytic leukemic cell line, THP-1, were purchased from the American Type Culture Collection (ATCC, USA). All cell lines were cultured under 37°C and 5% CO_2_ in appropriate medium supplemented with 10% FBS, 100 U/ml penicillin and 100 µg/ml streptomycin. THP-1 and B16 were maintained in RPMI-1640; 4T1 in DMEM.

### Flow Cytometric Analysis of Surface Marker Expression

After THP-1 cells were treated with AC (2 mg/ml) or PMA (10 ng/ml) for 72 hours, cells were harvested and fixed in 80% (v/v) ice-cold ethanol and kept at 4°C for 24 hours. The fixed cells were then washed and stained with 0.5 µg/ml PE-conjugated CD11b and CD14 antibody (BD Biosciences) in PBS containing 3% bovine serum albumin for 30 minutes at 4°C. The stained cells were washed twice in PBS containing 3% BSA and then fixed with 1% paraformaldehyde. The percentage of cells expressing the surface markers and the fluorescence intensity of the surface markers were analyzed by flow cytometric measurement conducted with a FACSCalibur cytometer (BD Biosciences).

### Analysis of Human Cytokine Array and Chemokine Array

Production of cytokines and chemokines by the AC-induced differentiated THP-1 cells were profiled using the Human Cytokine Array Kit (#ARY005, R&D system) and Human Chemokine Array Kit (#ARY017, R&D system). The cytokine array includes 36 cytokines and the chemokine array includes 37 chemokines (the full lists can be found at the R&D system website). THP-1 cells (1×10^6^ cells/well) were stimulated with 2 mg/ml AC extract for 48 hours. Then 1 ml of the cell-free supernatant was collected and incubated with the array membrane. The captured cytokines and chemokines were visualized by immunohistochemistry according to the manufacturer’s protocol.

### Statistical Analysis

All statistical analysis were carried out using the SPSS 13.0 statistical software package, and data were expressed as mean ± S.D. Student’s t-test was used for statistical comparisons of two groups. If the variances between groups were homogenous (Levene’s test), groups were subjected to the multiple comparisons with least significant differences (LSD) test. In case of no homogeneous variances, differences were evaluated by Welch’s T test and the groups were subjected to multiple comparisons with Dunnett’s T3 test. Survival curves were analyzed by a log-rank test. Statistical significance was set to p<0.05.

## Results and Discussion

### AC Extract Significantly Attenuated Tumor Growth and Prolonged Survival *in Vivo*

To determine and quantify the antitumor effect of the whole water extract of AC roots *in vivo*, we treated tumor-bearing mice (tumor developed from 4T1 cancer cells) with AC by gavage feeding for 21 days. We chose three AC doses for the study, i.e. 16 g/(kg·d) (high dose), 8 g/(kg·d) (medium dose) and 4 g/(kg·d) (low dose). Since the maximal solubility of AC in water is 1.6 g/ml, and we administered 10 ml/kg of the extract to the mice daily, the high dose used in our study, i.e. 16 g/(kg·d), was the maximal amount of AC extract that we could treat the mice with. Toxicology measurement confirmed that gavage feeding of 16 g/(kg·d) was safe and not causing any lethality *in vivo*. Figure 1A shows the tumor weight of the five treatment groups after 21 days, including: negative control group (Nctrl) treated with saline water; positive control group (Pctrl) treated with lentinan; high-dose AC treated group (hAC) at 16 g/(kg·d), medium-dose AC treated group (mAC) at 8 g/(kg·d) and low-dose AC treated group (lAC) at 4 g/(kg·d). We chose lentinan, a polysaccharide peptide isolated from a strain of Coriolus versicolor in China, as the positive treatment control, as lentinan is widely used as an immune-modulating anticancer remedy in China. It was found to enhance cell-mediated immune responses both *in vivo* and *in vitro*, and acts as biological response modifier that induces expression of various immuno-modulating cytokines [19], [20]. We observed no significant effect of AC treatment on blood counts or blood chemistry (Table S1 in File S1) of the tumor-bearing mice. Mice treated with all three doses of AC (i.e. low, medium and high) showed similar red blood cell count, white blood cell count as well as similar levels of alanine aminotransferase (ALT) and aspartate aminotransferase (AST) as those of the negative control group.

Tumor volumes of the different treatment groups were continuously monitored and mice treated with high-dose AC exhibited the slowest tumor growth (Table S2 in File S1). After 21 days of treatment, the average tumor weight in the high-dose AC treatment group was 96.7 mg, about 40% lower than that of the negative control group (160.7 mg), but the medium- and low-dose AC treatment groups did not show significant attenuation in tumor growth (Fig. 1A). Moreover, the degree of tumor size reduction observed in the high-dose AC treatment group was similar to that of the positive control group (average tumor weight of 107.4 mg), which convincingly confirmed the potent effect of high-dose AC in attenuating tumor growth *in vivo*.

Morphology of the tumor tissues from the five treatment groups was shown in Figure 1B. Compared to the negative control group, mice treated with medium- and high-dose of AC exhibited features associated with necrosis and infiltration of inflammatory cells in their tumor tissues, an indication of antitumor activity of the AC extract. In addition, we measured and compared the sizes of two important organs belonging to the immune system, i.e. spleen and thymus, in the different treatment groups. The size of the spleen and thymus relative to the mouse body weight was calculated and plotted in Figure 1C as spleen index and thymus index, respectively. While all treatment groups showed similar thymus index, medium- and high-dose AC treated mice showed a spleen index of 82.99 and 79.47, which was significantly higher than that of the negative control group (65.47) (*p*<0.01 or *p*<0.05). Given that AC treatment substantially increased the size of the spleen, we speculated that the water extract of AC may exert antitumor effect partly by enhancing the immune function of the spleen gland.

In addition to the effect on tumor growth, we also examined the potential antitumor activity of AC extract on prolonging the survival of melanoma-bearing mice. We used a well established and widely used mouse tumor model, the spontaneous C57BL/6-derived B16 melanoma, to evaluate the treatment effects of AC on survival [21], as advanced melanoma is known to be one of the most therapy-resistant cancer types. Figure 1D shows the cumulative survival curves of mice treated with AC extract in comparison with the control group that were treated with equal volume of saline water daily. The median survival time of the AC-treated group was 43 days, which was 1.6-fold longer than that of the control mice (27 days). Such strong effect on prolonging the lifespan of tumor-bearing mice, in particular for melanoma, further demonstrates that the whole water extract of AC has potent antitumor activity *in vivo*.

### Key Antitumor Cytokines were Induced in the AC-treated Mice

**Figure 2:**
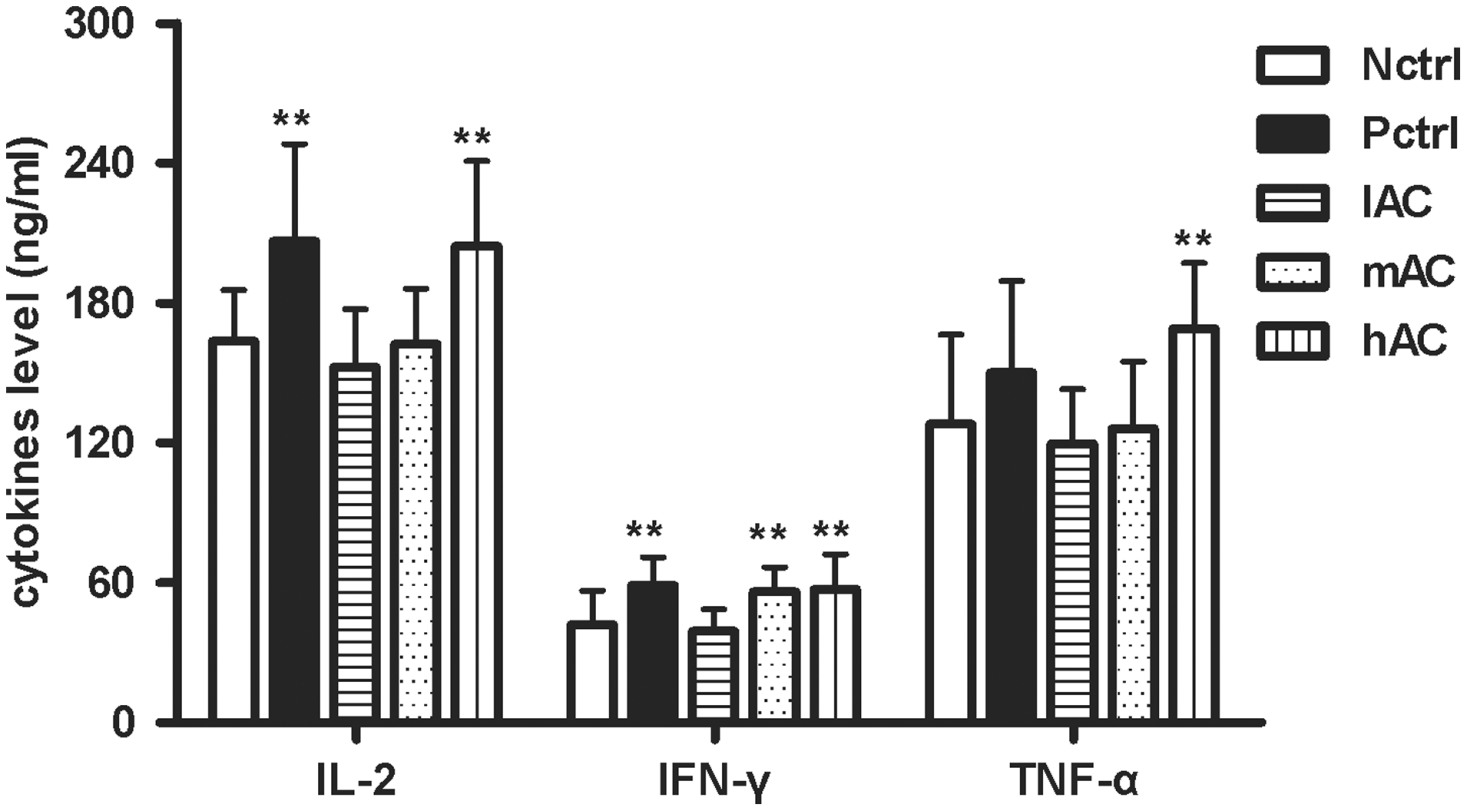
Levels of IL-2, INF-γ and TNF-α in the blood serum of the 4T1-bearing mice. Data are shown as mean ± S.D. averaged from nine or ten mice. Nctrl: negative control group; Pctrl: positive control group; lAC: low-dose AC treatment group; mAC: medium-dose AC treatment group; hAC: high-dose AC treatment group. **p<0.01 *vs* Nctrl group.

As we observed substantial increase in the spleen size of the AC-treated mice and that AC is traditionally used by Chinese medicine to modulate the immune balance [13], it is likely that one of the mechanisms by which AC exerts the strong antitumor effect *in vivo* is by enhancing the antitumor immune responses. To determine whether AC treatment induced release of cytokines that are associated with antitumor immunity, we measured by ELISA the levels of key cytokines, which have been shown to have important antitumor activity, in the blood serum of the 4T1 tumor-bearing mice under the five different treatment conditions as discussed above (Fig. 2). Our results showed that the levels of three cytokines were evidently increased under AC treatment, including IFN-γ, IL-2 and TNF-α. In particular, treatment of high- and medium-dose AC increased the serum level of IFN-γ as compared to the negative control group (p<0.01). Only the high-dose AC treatment significantly increased the release of IL-2 and TNF-α (p<0.01). These data suggest that AC extract, when used at high level, induced key antitumor cytokines that may further activate different immune cells and immune activity for attenuating tumor growth.

### AC Induced Differentiation of THP-1 Cells into Macrophage-like Cells

Although the mouse data indicated that AC may attenuate tumor growth by activating antitumor immunity, e.g. by increasing expression of antitumor cytokines, the cellular mechanism by which AC exerts its pharmacological effect is unresolved. Therefore, in addition to the mouse tumor models, we also investigated the effect of AC extract on cultured mammalian cells. We first measured the dose response of a panel of cultured cancer and normal cells to AC, including HeLa, U-2 OS, A549 and RPE. For all cell lines, we observed little cell death at all concentrations of AC that we used, with maximal dosage of 2 mg/ml. Most of the AC-treated cells continued to proliferate in cell culture. The lack of direct cytotoxicity of AC on culture cancer cells *in vitro* further indicates that AC may attenuate tumor growth and prolong survival by activating the antitumor immune response, instead of directly triggering tumor cell death.

**Figure 3:**
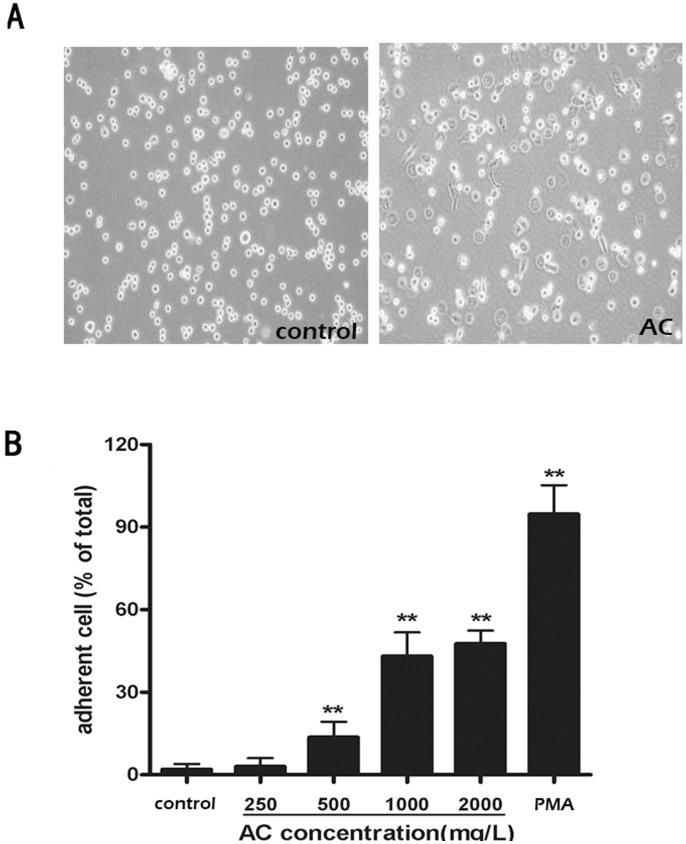
AC extract induced THP-1 differentiation in a dose-dependent manner. (A) Bright-field images of control THP-1 cells and THP-1 treated with 2 mg/ml AC extract for 72 hours. (B) Dose response of THP-1 cells to AC extract with respect to morpholgoical change from suspesion to adherence. Data were shown as mean ± S.D. averaged from three independent experiments. **p<0.01 *vs* control cells.

Antitumor immunity involves a wide variety of immune cells and a complex cascade of dynamic processes [22]. One of the key players in orchestrating the concerted immune response is macrophage [23]. To determine whether AC activates macrophage activity *in vitro*, we treated a human monocytic cell line, THP-1, with different doses of AC extract. As shown in Figure 3A, after 72 hours of AC treatment a significant number of THP1 cells changed from being in suspension to adherent (i.e. cell spreading and adhering to the culture dish) at AC concentrations ≥0.5 mg/ml. The morphological change from suspension to adherence is known to be a signature event when monocyte differentiates into macrophage.

We quantified the percentage of adherent cells under different concentrations of AC extract and compared the extent of AC-induced THP-1 differentiation with that under PMA, a potent activator of monocyte differentiation [24] (Fig. 3B). Our results showed that at saturating dosage of AC extract (2 mg/ml), about 48% of THP-1 cells differentiated to be adherent cells after 72 hours of treatment. Although the effect was substantial, AC extract was found less potent than PMA, which induced 95% of THP-1 cells to differentiate into macrophage-like cells after 24 hours of drug treatment.

### The Differentiated THP-1 Cells Induced by AC Expressed Surface Markers of Macrophage as Well as Antitumor Cytokines and Chemokines

**Figure 4:**
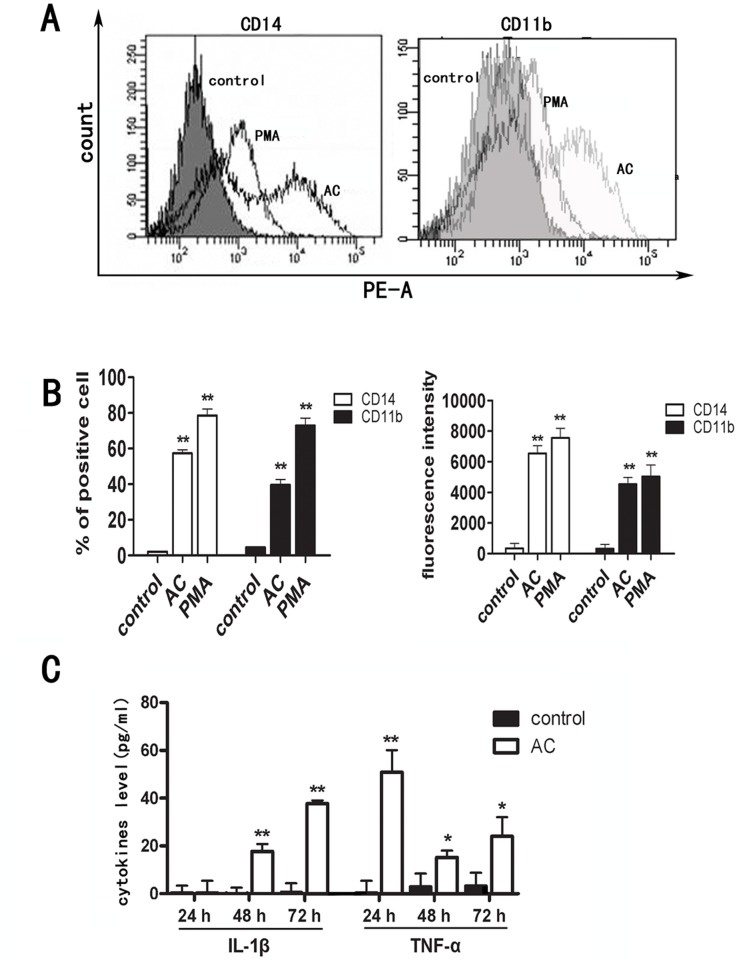
Characteristics of the differentiated THP-1 cells induced by AC. (A) Representative flow cytometric diagrams that showed the distributions of THP-1 cells with respect to expression of surface markers, CD14 and CD11b, under control (no treatment), PMA and AC treatment. (B) The ratio of surface marker positive THP-1 cells (left panel) and the fluorescence intensity of the surface markers (right panel) obtained from the flow cytometric diagrams. Data are shown as mean ± S.D. averaged from three independent experiments. *p<0.05, **p<0.01 vs control group. (C) ELISA analysis of IL-1β and TNF-α expression in THP-1 cells treated with AC at 2 mg/ml for 48 hours. Data are shown as mean ± S.D. averaged from two independent experiments. *p<0.05, **p<0.01 vs. control group.

To phenotype the differentiated THP-1 cells and determine whether they exhibited macrophage characteristics, we measured their expressions of surface markers specific to macrophage [25], including CD11b and CD14, by flow cytometric analysis. As shown in Figure 4A and 4B, after 72 hours of 2 mg/ml AC treatment the percentage of CD11b- and CD14-positive cells were 57% and 40%, respectively, in contrast to only 5% and 2% in non-treated THP-1. The mean fluorescence intensity of CD14 and CD11b were also significantly increased in the AC-treated cells compare to the control cells. These results suggest that the differentiated THP-1 induced by AC possessed key macrophage properties.

One way that the activated macrophages exert antitumor effect is by releasing specific cytokines, such as TNF-α and IL-1β. We thus measured the levels of TNF-α and IL-1β in the culture media of THP-1 cells treated with 2 mg/ml AC extract at selected time points by ELISA (Fig. 4C). Compared to the non-AC treated control THP-1, the culture media of the AC-treated cells showed high level of IL-1β (∼18 pg/ml) after 48 hours of treatment. Substantial increase in TNF-α level (∼51 pg/ml) was also observed under AC treatment and occurred faster, i.e. after 24 hours of AC treatment. Our data thus indicate that the AC-induced macrophages are capable of secreting antitumor cytokines, which may further activate the innate and adaptive immune responses against tumor.

**Figure 5:**
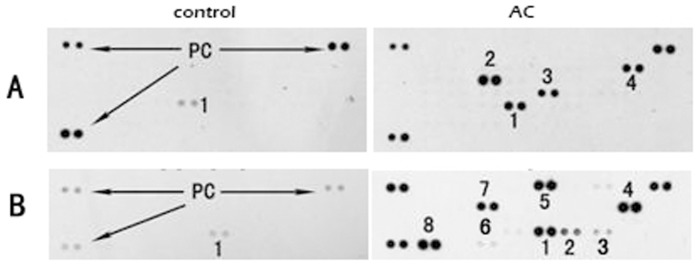
Expressions of human chemokines and cytokines from control THP-1 cells (control) vs. THP-1 treated with AC extract at 2 mg/ml for 48 hours (AC) (representative of three independent experiments). (A) Human chemokine array. PC, positive control; 1: RANTES; 2: IL-8/CXCL8; 3: MIP-1α, β; 4: GRO-α. (B) Human cytokine array. PC, positive control. 1: MIF; 2: MIP-1α; 3: MIP-1β; 4: IL-8; 5: GRO-α; 6: TNF-α; 7: IL-1ra; 8: RANTES.

To profile a broader range of cytokines and chemokines that may be produced by the differentiated THP-1 cells, we conducted cytokine and chemokine array analysis for a panel of 36 human cytokines and 37 human chemokines. Results from the array analysis showed that in addition to TNF-α and IL-1β, levels of Rantes, MIP-1α, MIP-1β, GRO-α, IL-8 and IL-1ra were also significantly elevated in the culture media of the differentiated THP-1 cells under AC treatment (Fig. 5). Rantes (CCL5), MIP-1α (CCL3), MIP-1β (CCL4) and GRO-α (CXCL1) are important chemokines that induce migration of different immune cells, such as T cells and monocytes [25], [26]. The array data thus suggest that the AC-induced macrophages may activate further antitumor immune response by attracting additional innate and adapted immune cells to the tumor site. As a variety of immune cells are known to be crucial components that constitute the tumor microenvironment and play indispensable roles in modulating the tumor physiology, a key mechanistic question to be addressed in further study is to identify the full cascade of immune responses as well as additional immune components that may be activated by the AC treatment.

## Conclusions

The root of *Alocasia Cucullata* (AC) has long been used for treating cancer by traditional Chinese medicine, but its pharmacological effects and mechanisms are poorly understood. In this study, using both mouse tumor models and cultured mammalian cells we not only confirmed the strong antitumor effects of the whole water extract of AC roots *in vivo* but also elucidated that a key mechanisms by which AC attenuates tumor growth and prolongs survival is by activating antitumor immunity, such as promoting the differentiation of monocytes into macrophages that are active against tumor. Our results thus provide important new insight for understanding not only the antitumor activities and immuno-modulating effects of AC but also the mechanistic basis to employ AC as a new, alternative anticancer treatment. However, in order to develop AC for broader clinical application, further, more detailed biochemical and pharmacological study is needed to determine both the active components of AC and the full spectrum of its biological activities.

## Supporting Information

File S1 **This file contains Tables S1, S2 and explanatory notes related to these two tables.**
(DOC)

## References

[1] Greenman C, Stephens P, Smith R, Dalgliesh GL, Hunter C, et al (2007) Patterns of somatic mutation in human cancer genomes. Nature 446: 153–158. 17344846 10.1038/nature05610 PMC2712719

[2] Sjöblom T, Jones S, Wood LD, Parsons DW, Lin J, et al (2006) The consensus coding sequences of human breast and colorectal cancers. Science 314: 268–274. 16959974 10.1126/science.1133427

[3] Lesterhuis WJ, Haanen JB, Punt CJ (2011) Cancer immunotherapy – revisited. Nat Rev Drug Discov 10: 591–600. 21804596 10.1038/nrd3500

[4] Coussens LM, Zitvogel L, Palucka AK (2013) Neutralizing tumor-promoting chronic inflammation: a magic bullet? Science 339: 286–291. 23329041 10.1126/science.1232227 PMC3591506

[5] Ghosh AK, Basu S (2012) Tumor macrophages as a target for Capsaicin mediated immunotherapy. Cancer Lett 324: 91–97. 22579786 10.1016/j.canlet.2012.05.002

[6] Chow KK, Naik S, Kakarla S, Brawley VS, Shaffer DR, et al (2013) T Cells Redirected to EphA2 for the Immunotherapy of Glioblastoma. Mol Ther 21: 629–637. 23070117 10.1038/mt.2012.210 PMC3589173

[7] Fiorenza S, Kenna TJ, Comerford I, McColl S, Steptoe RJ, et al (2012) A Combination of Local Inflammation and Central Memory T Cells Potentiates Immunotherapy in the Skin. J Immunol 189: 5622–5631. 23144496 10.4049/jimmunol.1200709 PMC3518562

[8] Ramakrishnan R, Gabrilovich DI (2011) Mechanism of synergistic effect of chemotherapy and immunotherapy of cancer. Cancer Immunol Immunother 60: 419–423. 20976448 10.1007/s00262-010-0930-1 PMC11029574

[9] Wettergren Y, Carlsson G, Odin E, Gustavsson B (2012) Pretherapeutic uracil and dihydrouracil levels of colorectal cancer patients are associated with sex and toxic side effects during adjuvant 5-fluorouracil-based chemotherapy. Cancer 118: 2935–2943. 22020693 10.1002/cncr.26595

[10] Gao Y, Tang W, Dai X, Gao H, Chen G, et al (2005) Effects of water-soluble Ganoderma lucidum polysaccharides on the immune functions of patients with advanced lung cancer. J Med Food 8: 159–168. 16117607 10.1089/jmf.2005.8.159

[11] Yang X, Huang S, Chen J, Song N, Wang L, et al (2010) Evaluation of the adjuvant properties of Astragalus membranaceus and Scutellaria baicalensis GEORGI in the immune protection induced by UV-attenuated Toxoplasma gondii in mouse models. Vaccine 28: 737–743. 19887128 10.1016/j.vaccine.2009.10.065

[12] Yoon TJ, Yu KW, Shin KS, Suh HJ (2008) Innate immune stimulation of exo-polymers prepared from Cordyceps sinensis by submerged culture. Appl Microbiol Biotechnol 80: 1087–1093. 18690428 10.1007/s00253-008-1607-y

[13] Hou X, Wu B, Zeng Z, Yong G (1998) Alocasia cucullata agglutinin purification and properties. Journal of South China Agriculatural University 19: 106–111.

[14] Kaur A, Kamboj SS, Singh J, Saxena AK, Dhuna V (2005) Isolation of a novel N-acetyl-D-lactosamine specific lectin from Alocasia cucullata (Schott.). Biotechnol Lett 27: 1815–1820. 16314976 10.1007/s10529-005-3559-y

[15] Lei X, Feng Y, Liang S, Wang Y, Zheng X (2012) Antitumor Effect and Chemical Constitutes of the Petroleum ether Fraction from the Rhizome of Alocasia cucullatta (Lour.) Schott. Chinese Journal of Pharmaceuticals 43: 340–343.

[16] Diesner SC, Wang XY, Jensen-Jarolim E, Untersmayr E, Gabor F (2012) Use of lectin-functionalized particles for oral immunotherapy. Ther Deliv. 3: 277–290. 10.4155/tde.11.146 PMC3572827 22834202

[17] Tundup S, Srivastava L, Harn DA Jr (2012) Polarization of host immune responses by helminth-expressed glycans. Ann N Y Acad Sci. 1253: E1–E13. 10.1111/j.1749-6632.2012.06618.x 22974465

[18] Megyeri P, Issekutz TB, Issekutz AC (1990) An endotoxin-induced factor distinct from interleukin-1 and tumour necrosis factor alpha produced by the THP-1 human macrophage line stimulates polymorphonuclear leukocyte infiltration in vivo. J Leukoc Biol 47: 70–78. 2403584 10.1002/jlb.47.1.70

[19] Ina K, Kataoka T, Ando T (2013) The use of Lentinan for Treating Gastric Cancer. Anti-cancer Agents in Medicinal Chemistry. Anticancer Agents Med Chem 13: 681–688. 23092289 10.2174/1871520611313050002 PMC3664515

[20] Yoshino S, Oka M (2008) Clinical trial of non-specific immunotherapy using Lentinan in advanced or recurrent gastric cancer. Gan To Kagaku Ryoho 35: 2239–2243. 19106583

[21] Overwijk WW, Restifo NP (2011) B16 as a mouse model for human melanoma. Curr Protoc Immunol. Chapter 20: Unit 20.1. 10.1002/0471142735.im2001s39 PMC2763508 18432774

[22] Candolfi M, Curtin JF, Yagiz K, Assi H, Wibowo MK, et al (2011) B cells are critical to T-cell-mediated antitumor immunity induced by a combined immune-stimulatory/conditionally cytotoxic therapy for glioblastoma. Neoplasia 13: 947–960. 22028620 10.1593/neo.11024 PMC3201571

[23] Guo J, Wang B, Zhang M, Chen T, Yu Y, et al (2002) Macrophage-derived chemokine gene transfer results in tumor regression in murine lung carcinoma model through efficient induction of antitumor immunity. Gene Ther 9: 793–803. 12040461 10.1038/sj.gt.3301688

[24] Daigneault M, Preston JA, Marriott HM, Whyte MK, Dockrell DH (2010) The identification of markers of macrophage differentiation in PMA-stimulated THP-1 cells and monocyte-derived macrophages. PLoS One 5: e8668. 20084270 10.1371/journal.pone.0008668 PMC2800192

[25] Zang YC, Samanta AK, Halder JB, Hong J, Tejada-Simon MV, et al (2000) Aberrant T cell migration toward RANTES and MIP-1 alpha in patients with multiple sclerosis. Overexpression of chemokine receptor CCR5. Brain 123: 1874–1882. 10960051 10.1093/brain/123.9.1874

[26] Zhu K, Shen Q, Ulrich M, Zheng M (2000) Human monocyte-derived dendritic cells expressing both chemotactic cytokines IL-8, MCP-1, RANTES and their receptors, and their selective migration to these chemokines. Chin Med J (Engl) 113: 1124–1128. 11776150

