# Supplementary material for: Alocasia cucullata Exhibits Strong Antitumor Effect In Vivo by Activating Antitumor Immunity
Source: PLoS One. 2013 Sep 25;8(9):e75328. doi: 10.1371/journal.pone.0075328 (PMC3783377; doi:10.1371/journal.pone.0075328)
Supplement: File S1 — This file contains Tables S1, S2 and explanatory notes related to these two tables. (DOC) [file pone.0075328.s001.doc]

Alocasia cucullata exhibits strong antitumor effect in vivo by activating antitumor immunity

Qiuxian Peng, Hongbing Cai, Xuegang Sun, Xin Li, Zhixian Mo ^*^ and Jue Shi ^*^

**Supporting Information File S1**

**Table S1:** Effects of AC treatment on blood counts and levels of ALT and AST in the tumor-bearing mice

| *group* | *RBC (×10^12^/L)* | *WBC (×10^9^/L)* | *ALT（U/L）* | *AST (U/L)* |
| --- | --- | --- | --- | --- |
| lAC | 5.72± 0.89 | 2.67 ± 0.92 | 15.68 ± 2.16 | 83.07 ± 12.25 |
| mAC | 5.46 ± 1.13 | 2.99 ± 0.18 | 14.46 ± 3.00 | 81.09 ± 10.38 |
| hAC | 5.56 ± 0.59 | 2.87 ± 0.78 | 15.26 ± 2.21 | 79.47 ± 9. 87 |
| Nctrl | 5.62 ± 0.74 | 2.64 ± 0.46 | 15.66 ± 2.31 | 61.47 ± 11.81 |
| Pctrl | 8.56 ± 1.19* | 3.77 ± 0.27* | 16.36 ± 3.37 | 80.76 ± 10.87 |

Data were reported as the mean ± SD (from 10 animals). RBC: red blood cell count; WBC: white blood cell count; ALT: alanine aminotransferase; AST: aspartate aminotransferase. lAC: low dose AC group; mAC: medium dose AC group; hAC: high dose AC group; Pctrl: positive control group (treated with lentinan); Nctrl: negative control group (treated with PBS). *p < 0.05, vs. Nctrl group.

**Table S2:** Tumor volumes (mm^3^) of mice in different treatment groups after the indicated days of treatment.

|  | Day 0 | Day 5 | Day 9 | Day 13 | Day 17 | Day 21 |
| --- | --- | --- | --- | --- | --- | --- |
| lAC | 19.7±20.3 | 29.8±24.9 | 68.5±39.6 | 90.3±44.0 | 123±77.4 | 156.5±74.3 |
| mAC | 21.5±20.5 | 36.0±33.8 | 74.0±41.1 | 85.9±37.9 | 103.3±28.5 | 144.8±57.0 |
| hAC | 25.2±30.4 | 27.0±33.8 | 39.6±41.0 | 43.3±37.9 | 60.5±28.5 | 86.3±32.0 |
| Nctrl | 23.7±31.7 | 27.7±31.4 | 84.7±68.8 | 106.8±70.5 | 145±72.7 | 181.8±69.7 |
| Pctrl | 17.9±20.0 | 24.2±19.2 | 28.0±19.7 | 69.3±31.4 | 82.4±23.9 | 116.1±42.2 |

Data were reported as the mean ± SD (from 10 animals).
